# Granular cell tumor presenting as an intraocular mass: a case report

**DOI:** 10.1186/s12886-019-1102-5

**Published:** 2019-04-25

**Authors:** Jingyuan Yang, Xinyu Ren, Youxin Chen

**Affiliations:** 1 Department of Ophthalmology, Peking Union Medical College Hospital, Peking Union Medical College, Chinese Academy of Medical Sciences, No.1 Shuaifuyuan, Wangfujing, Dongcheng District, Beijing, 100730 China; 2 Department of Pathology, Peking Union Medical College Hospital, Peking Union Medical College, Chinese Academy of Medical Sciences, No.1 Shuaifuyuan, Wangfujing, Dongcheng District, Beijing, 100730 China

**Keywords:** Granular cell tumor, Intraocular tumor, Milian body, Histopathologic examination

## Abstract

**Background:**

Granular cell tumor (GCT) can arise in any location in the body and present as a solitary, slow-growing, painless mass. However, GCT rarely affects the orbit. We report a Chinese girl who presented with an intraocular mass, and in whom a biopsy led to a diagnosis of GCT.

**Case presentation:**

A 5-year-old Chinese girl exhibited exotropia in her right eye for 2 years and had been losing her vision for 6 months. The visual acuity in the right eye revealed no light perception. A fundus examination showed a large, yellowish-white, elevated, subretinal mass lesion in front of and inferior to the disc, with hemi-inferior-quadrant retinal detachment. The retina was greyish-yellow with scattered yellow spots. A vitrectomy with neoplasm resection was performed. A histopathologic examination revealed a GCT. The tumor cells were positive for CD68, NSE, S-100, and CD163 expression but negative for GFAP, Syn, and CD123 expression. The Ki-67 index was 1%. The right eye remained stable with visual acuity of no light perception at a 2-years follow-up.

**Conclusion:**

Intraocular GCT can present as a yellow-white solid mass with no calcification. Although intraocular GCT is very rare, it can lead to devastating visual loss. Intraocular GCT should be kept in mind and considered in clinical practice.

## Background

Granular cell tumor (GCT) is a rare neoplasm commonly observed in the head and neck, however, it can also occur in various other locations, such as the skin, gastrointestinal tract, and central nervous system [1]. It usually occurs as a solitary, slow-growing, painless mass and is frequently diagnosed as an incidental finding on biopsy. To our knowledge, orbital masses caused by GCT account for approximately 3% of all GCT cases but have not been reported in eye balls [2]. Here, we reported a Chinese girl in whom unilateral exotropia and blindness were the initial symptoms on presentation to the ophthalmologist. A subsequent biopsy provided a convincing the diagnosis of GCT.

## Case presentation

**Fig. 1:**
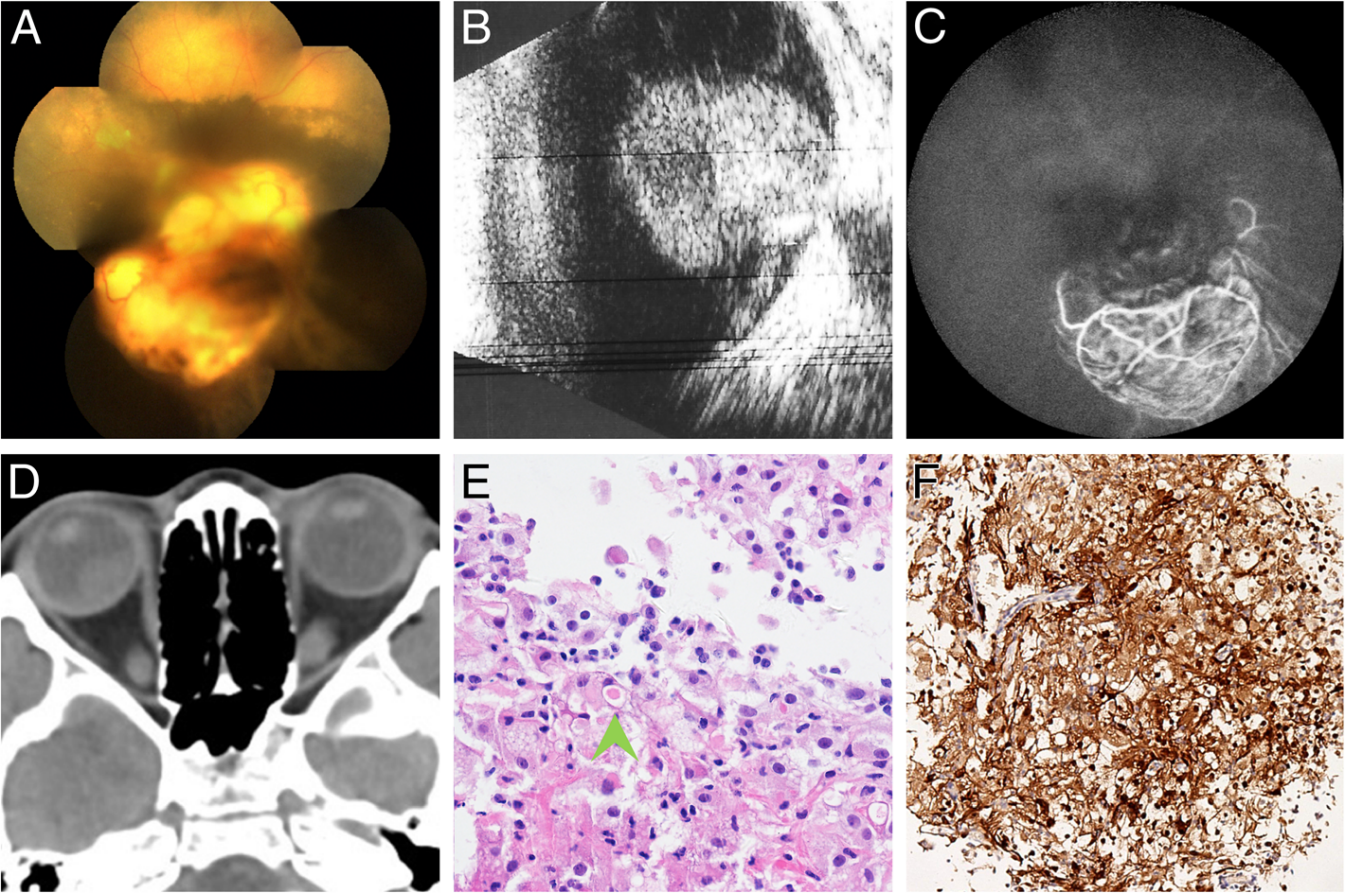
**a** Color photograph shows a yellowish-white, elevated, subretinal mass lesion, with hemi-inferior-quadrant retinal detachment. **b** B-scan ultrasonography shows an intraocular solid mass with moderate-to-high internal reflectivity located in front of the disc, and no calcification was noticed. **c** Fluorescein angiogram shows double circulation and mottled fluorescence on the mass. The retina was out of focus and fuzzy. **d** Computed tomography showed a semi-round, slightly high-density shadow in the right eye. **e** A pustulo-ovoid body of Milian was observed (green arrowhead) (haematoxylin-eosin, original magnification × 40). **f** Positive immunostaining for S-100 was confirmed

A 5-year-old Chinese girl who had exhibited sensory exotropia in her right eye for 2 years and had been losing her vision for 6 months was referred to our department. There was no history of postnatal asphyxia and no family history of tumors, or other ocular disorders. A general examination of the child revealed no other abnormalities. On examination, the visual acuity in the right eye revealed no light perception and an intraocular pressure of 7 mmHg. The anterior chamber reaction and pigment cells in the vitreous were observed. A fundus examination showed a large, yellowish-white, elevated, subretinal mass lesion in front of and inferior to the disc that showed hemi-inferior-quadrant retinal detachment (Fig. 1a). The retina was greyish-yellow with scattered yellow spots. Examination of the left eye showed no abnormalities. B-scan ultrasonography and fluorescein angiography (FA) of the right eye revealed an intraocular solid mass located in front of the disc (Fig. 1b). The mass measured 11.0 mm in diameter and had moderate-to-high internal reflectivity, a distinctive border and no calcification on B-scan ultrasonography. Fundus fluorescein angiography showed double circulation and mottled fluorescence on the mass, with no obvious leakage (Fig. 1c). Computed tomography of the orbit revealed a semi-round, slightly high-density shadow with a CT value of approximately 46 Hu (Fig. 1d). Then, a vitreous biopsy for tumor cells produced negative results. After 9 months, the parents of the child agreed to further diagnosis and treatment, and a vitrectomy with lensectomy and neoplasm resection with silicone oil tamponade were performed to achieve a histopathological examination. The tough mass had a distinctive border and no obvious capsule and showed no involvement of the extraocular muscles, optic nerve or orbital tissues. Histopathologic examination of the intraocular mass revealed a GCT (Fig. 1e). The tumor cells were positive for CD68, NSE, S-100 (Fig. 1f), and CD163 expression but negative for GFAP, Syn, and CD123 expression. The Ki-67 index was 1%, which strongly suggested that this tumor was benign. At the last follow-up, which was performed more than 2 years after the first visit, no GCT recurrence was noted and the right eye remained stable, but with permanent blindness eventually.

## Discussion and conclusion

Orbital GCT usually presents as a local mass in the lower half of the orbit (58.3%) that shows inferior rectus (38.5%) involvement [2]. In these patients, diplopia is a prominent symptom and is usually accompanied by progressive proptosis due to the slow-growing painless mass. Ribeiro et al. found in a literature survey that there were only 39 such cases reported in the literature, and they revealed that orbital GCT rarely affects the orbit [2]. To our knowledge, this case is the first report of an intraocular GCT that was confirmed by biopsy and was challenging due to its location.

We first described the clinical features and visual outcomes of this intraocular GCT. Due to a lack of knowledge of intraocular GCT, it is very difficult for ophthalmologists to make a diagnosis of intraocular GCT, even in patients with an intraocular mass that can be seen in vivo directly through a dilated pupil. From the present patient, we learned that intraocular GCTs can mimic other tumors, including retinoblastoma, and can be negative on vitreous biopsy. Intraocular GCT, a newly discovered intraocular tumor, should also be also considered when diagnosing an intraocular mass.

A diagnosis of GCT is made based on a histopathologic examination that shows characteristically abundant eosinophilic granular cytoplasm and Milian bodies [3]. Immunohistochemical and electron microscopic studies have recently indicated that GCT represents a neural Schwann cell-related neoplasm [4, 5]. Interestingly, the origin of the intraocular GCT in the present case is unknown because the neuroretina had no myelin sheath, and the myelin sheath of the optic nerve had no Schwann cells. One hypothesis that has been endorsed by some authors, including us, is that these tumors are biologically heterogeneous in spite of their histological homogeneity [6–8].

The present case showed no existing malignant criteria in part because of the small size of the specimen [1, 9]. However, the Ki-67 index value of 1%, which has also been used by other ophthalmologists, [9, 10] strongly suggests that the present GCT was a benign GCT.

Because multiple or multifocal GCTs have been reported in up to approximately 25% of cases, [1] and because intraocular GCTs can lead to blindness secondary to retinal detachment, we recommend that patients with GCTs should be referred to an ophthalmologist and propose that such recommendations can potentially save the patients’s ability to see light. In addition, in such a challenging situation in which there are no published guidelines, protocols, or clinical trials available to select GCT treatments, tumor excision rather than chemotherapy or radiotherapy is currently recommended for almost all GCTs, similar to recommendations for those occurring in other sites throughout the body [1].

In conclusion, although intraocular GCTs are very rare, they can lead to devastating visual loss. The most relevant finding in this report is that intraocular GCTs should be kept in mind and considered in clinical practice in patients presenting with an intraocular mass and patients with GCTS. We report this rare case so that ophthalmologists who see patients with GCTs can be made more aware of the potential ophthalmic risks including blindness.

## Abbreviation

GCT: Granular cell tumor
